# Effects of cognitive behavioral therapy on improving depressive symptoms and increasing adherence to antiretroviral medication in people with HIV

**DOI:** 10.3389/fpsyt.2022.990994

**Published:** 2022-11-09

**Authors:** Keke Qin, Jiale Zeng, Li Liu, Yumei Cai

**Affiliations:** ^1^School of Politics and Public Administration, Guangxi Normal University, Guilin, China; ^2^School of Management, Jinan University, Guangzhou, China; ^3^School of Social and Public Administration, East China University of Science and Technology, Shanghai, China; ^4^Population Research Institute, Peking University, Beijing, China

**Keywords:** cognitive behavioral therapy, Human Immunodeficiency Virus, depression, medication adherence, meta-analysis

## Abstract

The incidence of depression is higher in PLWH (people living with HIV) than in the general population. It is of clinical significance to explore effective measures to improve depression in patients. But the available evidence is still quite limited. CBT (cognitive behavioral therapy) is considered to be one of the effective methods to improve depression, medication adherence and quality of life in PLWH. Therefore, this study aimed to systematically evaluate the effect of cognitive behavioral therapy on improving depressive symptoms and increasing adherence to antiretroviral therapy (ART) in people living with HIV (Human Immunodeficiency Virus). The Cochrane Library, Embase, PubMed, and Web of Science databases were searched by computer to collect randomized controlled trials on the effects of cognitive behavioral therapy on improving depression and increasing ART medication adherence in PLWH, and the retrieval time was from the inception of each database to January 10, 2022. Meta-analysis was performed by two researchers using Stata 15.0 software after screening the literature, extracting data and evaluating quality according to inclusion and exclusion criteria. A total of 16 studies with 1,998 patients were included. Meta-analysis results showed that CBT improved depressive symptoms in PLWH (SMD = −0.09, 95% CI [−0.13 to −0.04], *P* < 0.001) with better long-term (<6 months) depression improvement (SMD = −0.09, 95% CI [−0.15 to −0.02], *P* = 0.006) than short-term (0–6 months); the difference in improved ART medication adherence in the CBT group compared to the control group was not statistically significant (SMD = 0.04, 95% CI [−0.06 to 0.13], *P* = 0.490). There may be publication bias due to incomplete inclusion of literature as only published literature was searched. Cognitive behavioral therapy is effective in improving depressive symptoms in people living with HIV, with better long-term (>6 months) results than short-term (0–6 months).

## Introduction

According to the World Health Organization, ~37.7 million people (95% CI, 30.2–45.1 million) are infected with HIV (World Health Organization) (1). People living with HIV (PLWH) are more likely to suffer from mental health disorders than the general population (2). The incidence of depression is higher in PLWH than in the general population, and depression decreases ART medication adherence in PLWH which is key to the success of highly active antiretroviral therapy (HAART) (3, 4). Some studies have shown that only 95% or more adherence to treatment can ensure the effectiveness of antiviral therapy (5, 6). However, adherence to medication among people living with HIV is not encouraging. A cross-sectional survey in Latin America showed that the overall adherence rate for PLWH was only 70% (7). It has also been documented that only 67.51% of PLWH achieve 100% medication adherence (8). Therefore, effective measures need to be explored to improve patients' depressive symptoms and promote ART medication adherence.

Given the high prevalence of depression in PLWH and its negative effects, more strategies and interventions have been implemented, such as medication and psychotherapy. Studies have found a significant correlation between depressed mood and treatment adherence, quality of life, and life expectancy in PLWH (9, 10). Therefore, psychological interventions in the treatment of depression in PLWH play a key role in the treatment of depression in PLWH. In recent years, CBT has been widely used by many researchers in the management of mental health of PLWH. CBT has been shown to be effective in improving adherence, depressive symptoms, stress, and quality of life in PLWH IDUs (injecting drug users), MSM (men who have sex with men), etc., (11–14) and more effective in reducing depressive symptoms and increasing ART medication adherence than other psychological intervention models (15). Therefore, this study examines the effect of CBT on the remission of depressive symptoms and adherence to ART medication in PLWH.

Cognitive behavioral therapy (CBT) is one of the methods of psychotherapy, which believes that irrational cognition and behavior can affect emotions and cause or exacerbate psychological and physical problems. CBT is a structured, problem-centered treatment approach that encompasses a wide range of interventions, making it difficult to give a single and clear definition. According to Christopher, CBT is based firstly on the idea that thoughts affect the individual's emotions and behaviors in response to events that occur; secondly, perceptions and interpretations of events are shaped by the individual's beliefs and assumptions; logical fallacies and cognitive distortions can occur in people experiencing psychological distress; CBT can modify the patient's bad thoughts and thus reduce psychopathology (16). Based on the above concepts, this meta-analysis defines CBT as follows: (1) PLWH (people living with HIV)associate their symptoms with distress, irrational thoughts and beliefs, and behaviors; (2) reassessment of cognitions, beliefs, or reasoning related to the target symptoms; (3) psychological interventions independent of other therapeutic measures, such as challenging habitual thinking patterns, listing evidence of false beliefs, giving alternative plausible and individualized explanations based on reasoning ability and personal experience and testing them in reality; (4) either case therapy or group therapy is available.

Currently, no evidence-based studies with a high level of evidence have been conducted on the effects of CBT on improving depression and increasing ART medication adherence in PLWH in spite of several studies on the effects of CBT on depression and medication adherence in PLWH. Much of the available evidence focuses on specific populations of people living with HIV, with few reports of systematic evaluation of people living with HIV as a whole. For example, one systematic review has focused on psychological interventions, including cognitive behavioral therapy for PLWH with common psychiatric disorders such as depression and anxiety. However, this review only presented the characteristics of the five included studies which were not processed by meta-analysis (17). Another meta-analysis focused on the intervention of CBT on depressive symptoms in PLWH, revealing the short-term effectiveness of CBT on depressive symptoms in PLWH (18). Studies have shown that a series of interventions through CBT can improve depression and quality of life for PLWH, with the best results for those in the latent phase of the disease (19). As for multiple studies on specific populations of people living with HIV, for instance, a systematic review of cognitive behavioral therapy to improve mental health in women with AIDS (Acquired Immune Deficiency Syndrome) including three studies measuring mental health QOL (quality of life) and stress demonstrated statistically significant improvement in reducing psychological distress, improving symptoms of depression, stress and QOL (15). Nevertheless, the efficacy of CBT for people living with HIV/AIDS has been predominantly characterized in men who have sex with men and was more pronounced when participants possessed higher education and treatment providers used cognitive behavioral therapy (CBT) (20, 21). Group-based psychosocial interventions based on cognitive behavioral therapy (CBT) may have a small effect on measures of depression, and this effect may last for up to 15 months after participation in the group sessions, but the clinical importance of this is unclear (22). Studies have also shown improvement in depressive symptoms among people living with HIV/AIDS in low- and middle-income countries (23). Taken together, the available evidence is still quite limited. The aim of this study was to systematically evaluate studies on depressive symptoms and ART medication adherence in PLWH so as to provide evidence-based medical evidence for CBT intervention in PLWH.

## Methods

### Literature search

Computer searches of the Cochrane Library, Embase, PubMed, and Web of Science databases were conducted to collect randomized controlled trials (RCTs) on the effects of cognitive behavioral therapy on improving depression and increasing medication adherence in PLWH, with the search time set from the inception of each database to January 10, 2022. For PubMed, the search strategy was to retrieve articles containing the terms PLWH, CBT, and RCT in the subject terms and free terms. A similar strategy was used when searching other databases Taking PubMed as an example, the specific search strategy is shown in Appendix.

### Inclusion and exclusion criteria

- Studies that met the following inclusion criteria were included in the meta-analysis.
- Subjects: patients aged 18 years or older with a confirmed HIV-positive diagnosis, including HIV-infected patients and patients with AIDS.
- Study design: randomized controlled trial (RCT) with no language restrictions.
- Interventions: the CBT group received conventional nursing or other psychological intervention plus CBT. The control group used conventional nursing or other psychological intervention models.
- Outcome measures: (1) Depression: the Beck Depression Inventory (BDI), Hamilton Depression Scale (HAMD), Center for Epidemiological Survey-Depression Scale (CES-D), Profile of Mood States (POMS), the Clinical Global Impression (CGI), and the Montgomery-Asberg Depression Rating Scale (MADRS) were used for assessment. (2) ART medication adherence: self-report, the Medication Event Monitoring System (MEMS), the Simplified Medication Adherence Questionnaire (SMAQ), and Wispill Technologies were used for evaluation.

Exclusion criteria for meta-analysis were as follows:

- Literature reviews, systematic reviews, case reports, or study protocols.
- Studies unrelated to the topic.
- Non-CBT research interventions.
- Study subjects without PLWH or depression.

### Data extraction

Each included study was coded by two researchers and the Excel spreadsheet was completed according to the following details: author, year of publication, country, participant characteristics, specific descriptions of the intervention and control groups, etc. Data were independently extracted and cross-checked by two investigators according to the inclusion and exclusion criteria. The extracted information included study subjects, interventions, control measures, intervention duration, follow-up duration, outcome indicators, and evaluation time of outcome indicators. All included studies were independently evaluated and differences were discussed until consensus was reached.

### Quality assessment

Two investigators independently screened the literature according to the inclusion and exclusion criteria, extracted and cross-checked the data, and assessed the literature quality. The risk of bias assessment tool for systematic reviews of interventions from the Cochran Collaborative (version 5.1.0) (24) was adopted to evaluate the methodological quality of the included studies.

### Data analysis

Meta-analysis was performed using Stata 15.0 software. For continuous data, the mean difference (MD) was used as the effect analysis statistic if the results were obtained using the same measurement tool, and the standardized mean difference (SMD) was used as the effect analysis statistic if different measurement tools were employed for the same variables. Chi-square test was adopted to determine whether there was statistical heterogeneity among the results. If *P* ≥ 0.1 and *I*^2^ < 50%, multiple similar studies were considered to be homogenous, and a fixed-effects model was used for meta-analysis. If *P* < 0.1 and *I*^2^ ≥ 50% with clinical homogeneity, a random-effects model was selected.

**Figure 1:**
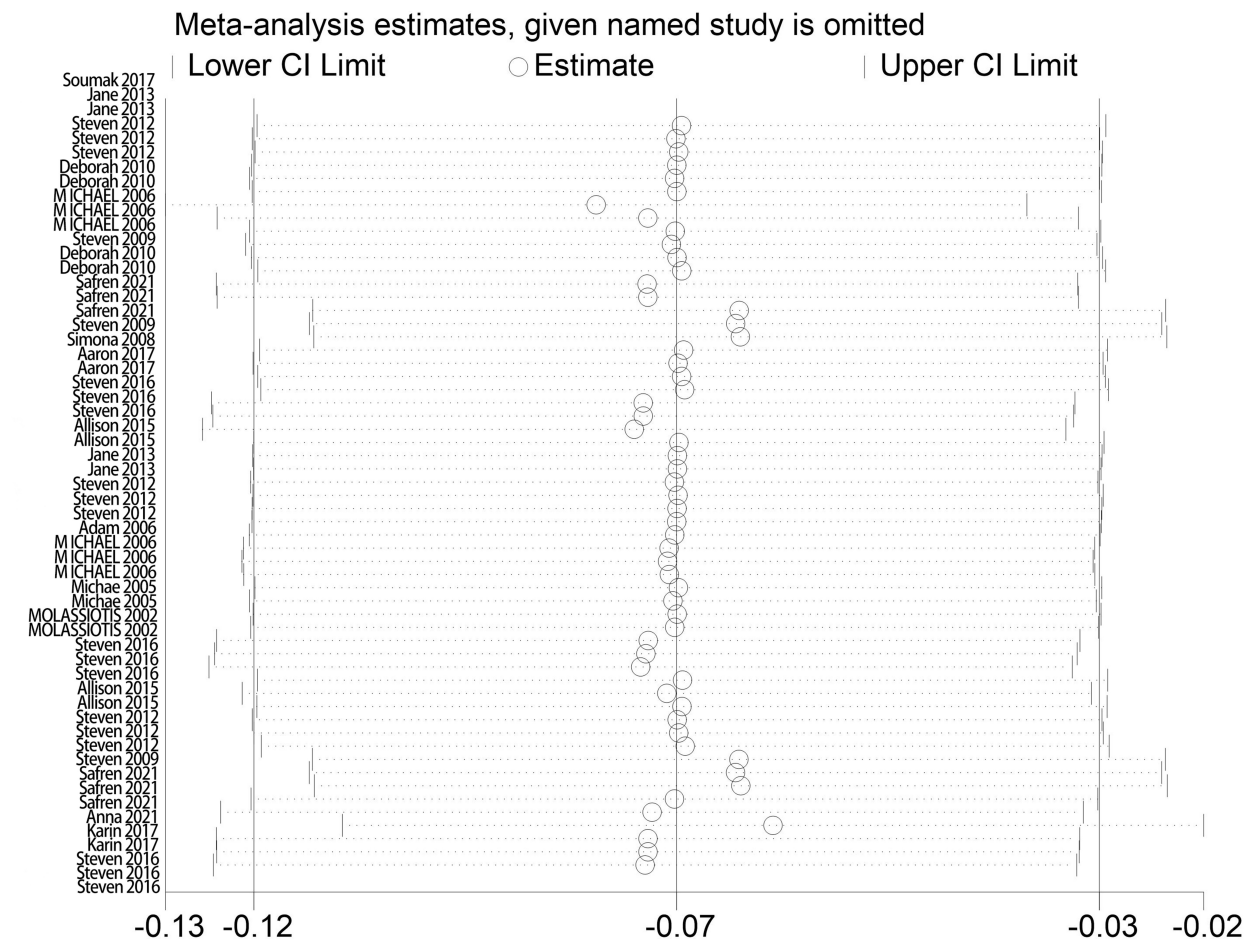
Sensitivity analysis of depressive symptoms.

**Figure 2:**
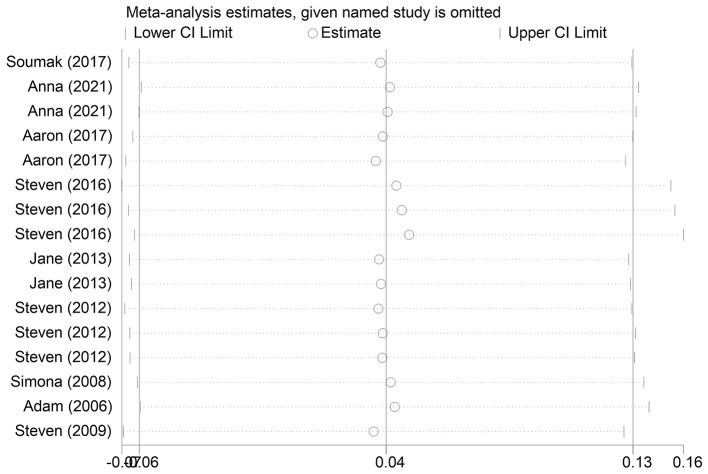
Compliance sensitivity analysis.

**Figure 3:**
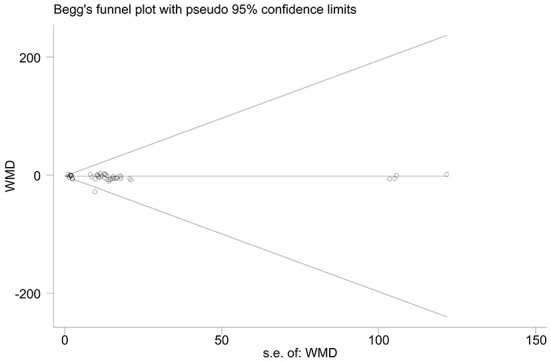
Funnel plot of depressive symptoms.

**Figure 4:**
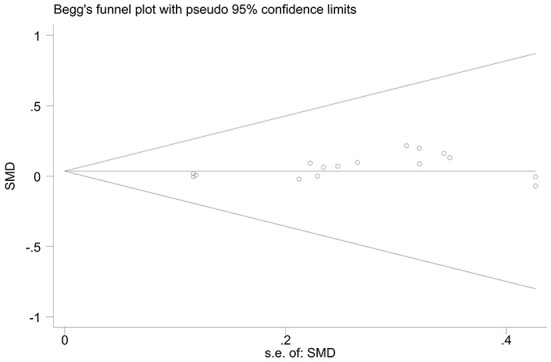
Funnel plot of adherence.

The effect extent of the combined effect size on depressive symptoms and ART medication adherence and the robustness of the results were explored by the one-by-one exclusion method. No significant changes in the results occurred after the exclusion of the two study groups, indicating low sensitivity and relatively robust and reliable results (Figures 1, 2). Begg's test was used to detect the publication bias of the two groups. In the depressive symptoms group, *P* = 0. 0.107 for Begg's test, and thus the publication bias was excluded (Figure 3); *P* = 0.065 for Begg's test in the ART medication adherence group, thereby excluding the publication bias (Figure 4).

### Subgroup analysis grouping

On depressive symptoms, some literature has shown differences of CBT in long-term and short-term interventions for depressive symptoms in PLWH populations (25). Studies also have demonstrated high heterogeneity between different depression scales so that the use of intervention duration and different scales as a basis for subgroup analysis grouping in subgroup analysis needs to be introduced (26). In terms of ART medication adherence, the results of some studies have shown that the duration of follow-up in PLWH is directly related to patients' tolerance of the medication and adherence to regular medication (27), and thus subgroups of length of follow-up will be used to analyze ART medication adherence in PLWH in order to observe the extent to which length of follow-up affects medication adherence.

## Results

### Study selection

**Figure 5:**
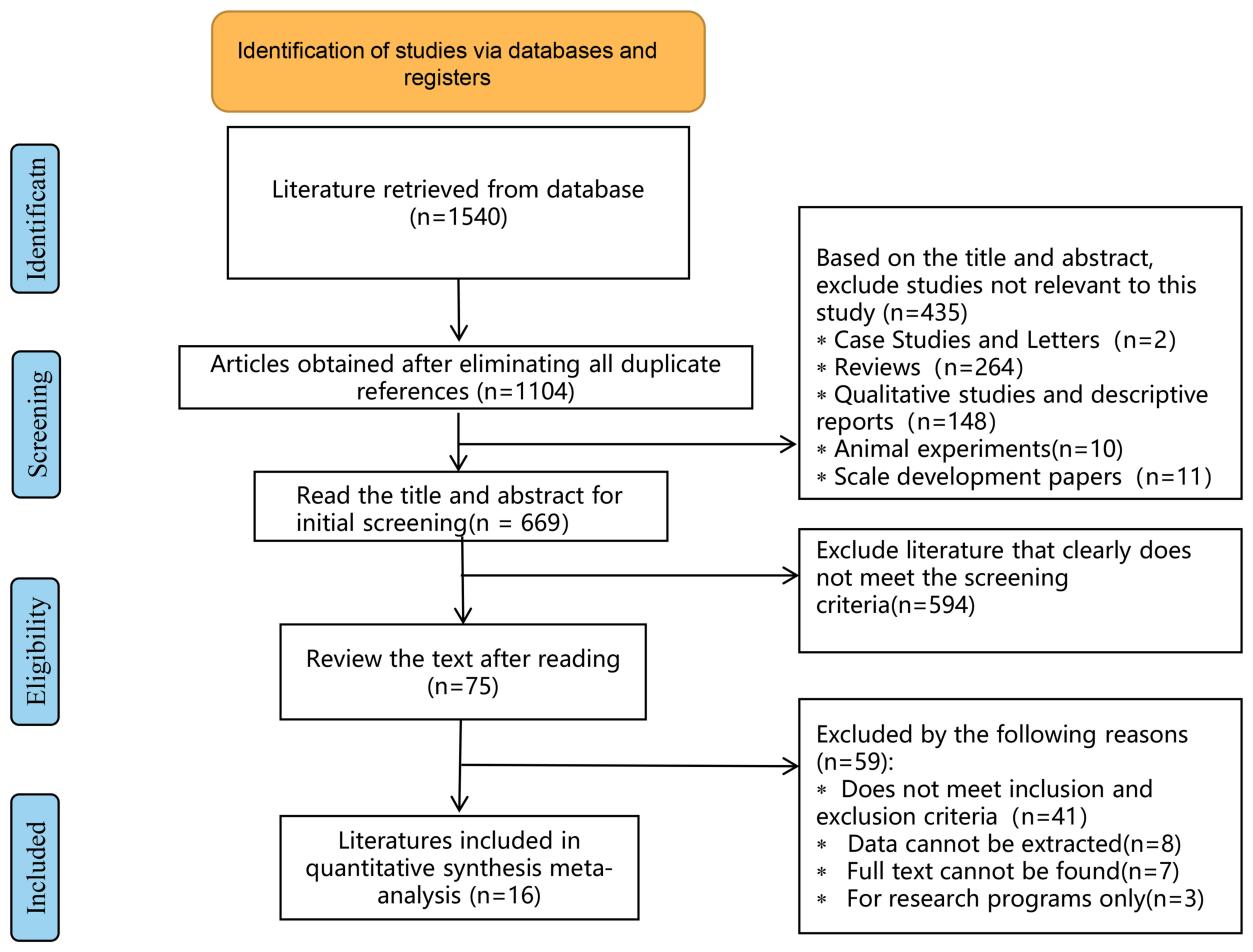
Flow diagram.

A total of 1,540 relevant articles were initially retrieved, and after stratification screening, 16 studies (13, 28–42) with a total of 1,998 patients were finally included. The literature screening process and results are shown in Figure 5. The 16 studies enrolled 1,998 patients from the United States, Australia, China, Nigeria, India, and Switzerland. The CBT group received 6 to 12 times of intervention, with each intervention lasting from 30 to 150 min. The duration of intervention lasted varied from 6 weeks to 4 months, and the follow-up lasted from 3 months to 36 weeks. The basic information of the included studies is detailed in Table 1, and the methodological quality evaluation of the included studies is shown in Table 2.

**Table 1:** Basic characteristics of included studies.

**Author**	**Year**	**Country**	**Group**	**Samplesize**	**Detail**	**Outcome indicator assessment time**	**Outcome**
Safren	2021	USA	CBT-AD	80	Seven interventions, once a week, were intensified 9 months after the end of treatment	4, 8, and 12-month	Depression:
			Control (ETAU)	81	The patients were mainly treated with medication and returned to the hospital once a month	4, 8, and 13-month	
Anna	2021	USA	CBT-AD	11	10–12 weeks of treatment, once a week	3 and 6-month	Depression: Adherence:
			Control (SP)	11	Drug therapy mainly supplemented by drug control strategy	3 and 6-month	
Karin	2017	Australia	CBT	162	Ten 90-min sessions over 5 weeks, one individual intervention and nine group interventions	6 and 12-month	Depression:
			Control	153	1 group meeting	6 and 12-month	
Soumak	2017	India	CBT	30	Eight 30-min interventions were performed over a 6-month period	6-month	Depression: Adherence:
			Control	30	Psychological education and supportive counseling	6-month	
Aaron	2017	USA	CBT-BISC	22	Twelve interventions were performed once a week for 50 min over 3 months	3 and 6-month	Depression: Adherence:
			Control (ETAU)	22	Six meetings in 3 months, 15 min every 2 weeks	3 and 6-month	
Steven	2016	USA	CBT-AD	94	11-session counseling modules, each lasting up to 60 min	4, 8, and 12-month	Depression: Adherence:
			Control 1 (ISP)	97	A single-session adherence counseling intervention following the Life-Steps	4, 8, and 13-month	
			Control 2 (ETAU)	49	Psychological intervention conversations lasted 60 min each	4, 8, and 14-month	
Allison	2015	USA	CBT-AD	41	Eight separate treatments	3 and 12-month	Depression:
			Control (ETAU)	40	Drug intervention	3 and 12-month	
Jane	2013	USA	CBT-AD	20	Nine treatments of 50 min were given every 1–2 weeks for 4 months	6 and 9-month	Depression: Adherence:
			Control (TAU)	20	Alarmed pillbox or usual care	6 and 9-month	
Steven	2012	USA	CBT-AD	44	Eight treatments, each lasting ~50 min, were performed weekly, with the goal to be achieved in ~3 months	3, 6, and 12-month	Depression: Adherence:
			Control (ETAU)	45	Nine general psychological counseling sessions	3, 6, and 12-month	
Deborah	2010	USA	CBSM	212	This condition was a 10-week manualized group therapy program of weekly 120-min sessions (90-min cognitive-behavioral stress management, 30-min relaxation) with encouragement to practice between sessions.	Post and 3-month; 6 and 12-month	Depression:
			Control	212	The comparison condition consisted of 10-weekly individual 120-min information sessions (45-min informational/educational videotape component supplemented by a 75-min entertainment video tape).	Post and 3-month; 6 and 12-month	
Steven	2009	USA	CBT-AD	23	10–12 times intervention	3, 6, and 12-month	Depression: Adherence:
			Control (ETAU)	22	A single-session intervention for adherence and a letter to the patient's provider documenting her or his continued depression.	3, 6, and 12-month	
Simona	2008	Switzerland	CBSM	53	Training consisted of 12 weekly group sessions lasting 2 h and provided during a 12 week period for each group.	12 months	Depression: Adherence:
			Control	51	A visit lasted, on average, ~30 min	12 months	
Adam	2006	USA	CBSM+MAT	76	Participants attended weekly 135-min sessions (90-min stressmanagementand 45-min relaxation) and were instructed to practice relaxation exercises twice daily between sessions, lasts for 10 weeks	10 weeks	Depression: Adherence:
			Control (MAT)	54	A 1-h session at baseline as well as two 30-min maintenance sessions at 3 and 9 months post-randomization, respectively	10 weeks	
M ICHAEL	2006	USA	CBSM+MAT	76	10 weekly 135-min group sessions (90 min stress management and 45 min relaxation) and were instructed to practice relaxation exercises twice daily between sessions.	3, 6, and 15-month	Depression:
			Control (MAT)	54	1-h session at baseline, as well as half-hour maintenance sessions at 3 and 9 months post-randomization, respectively.	3, 6, and 15-month	
Michael	2005	USA	CBSM	16	10 weekly 135-min sessions (45-min relaxation component and 90-min stress management component) and were instructed to practice relaxation exercises twice daily between sessions.	Post-CBSM; 6–12-month	Depression:
			Control	9	10 weeks of general training. After the 10-week post-treatment assessment, they were offered a 1-day stress management workshop summarizing several of the concepts presented in the CBSM sessions.	Post-CBSM; 6–12-month	
MOLASSIOTIS	2002	HK	CBT	36	Once a week for 12 weeks	Pre-intervention; Post-intervention; 3 month	Depression:
			Control 1 (PSC)	26	In relation to improving mood and quality of life and decreasing uncertainty in illness as compared to a group receiving routine treatment with no formal psychosocial intervention.	Pre-intervention; Post-intervention; 3 months	
			Control 2 (Comparison)	26	Ntervention or treatment as usual	Pre-intervention; Post-intervention; 3-month	

Depression: Beck Depression Inventory, BDI; Hamilton Depression Scale, HAMD; Center for Epidemiologi-cal Survey-depression Scale, CES-D; Profile of Mood States, POMS; The ClinicalGlobal Impression, CGI; Montgom-ery-Asberg Depression Rating Scale, MADRS; adherence: Self-report; Medication Event Monitoring System, MEMS; The Simplified Medication Adherence Questionnaire (SMAQ); Wispill Technologies.

**Table 2:** Methodological quality assessment.

**Author**	**Year**	**Random sequence generation (selection bias)**	**Allocation concealment (selection bias)**	**Blinding of participants and personnel (performance bias)**	**Blinding of outcome assessment (detection bias)**	**Incomplete outcome data (attrition bias)**	**Selective reporting (reporting bias)**	**Other bias**
Safren	2021	Low risk	Unclear	Low Risk	Low risk	Low Risk	Low risk	Low risk
Anna	2021	Low risk	Unclear	Unclear	Unclear	High risk	Low risk	Low risk
Karin	2017	Low risk	Unclear	Unclear	Unclear	Low risk	Low risk	Low risk
Soumak	2017	Low risk	Unclear	Unclear	Unclear	Low risk	Low risk	Low risk
Aaron	2017	Low risk	Unclear	Low risk	Low risk	Low risk	Low risk	Low risk
Steven	2016	Low risk	Unclear	Low risk	Low risk	Low risk	Low risk	Low risk
Allison	2015	Low risk	Unclear	Unclear	Unclear	Low risk	Low risk	Low risk
Jane	2013	Low risk	Low risk	Low risk	Low risk	Low risk	Low risk	Low risk
Steven	2012	Low risk	Unclear	Low risk	Low risk	Low risk	Low risk	Low risk
Deborah	2010	Low risk	Unclear	Unclear	Unclear	Low risk	Low risk	Low risk
Steven	2009	Low risk	Low risk	Low risk	Low risk	Low risk	Low risk	Low risk
Simona	2008	Low risk	Low risk	Low risk	Low risk	Low risk	Low risk	Low risk
Adam	2006	Low risk	Unclear	Low risk	Unclear	Low risk	Low risk	Low risk
M ICHAEL	2006	Low risk	Unclear	Unclear	Unclear	Low risk	Low risk	Low risk
Michae	2005	Low risk	Unclear	Unclear	Unclear	Low risk	Low risk	Low risk
MOLASSIOTIS	2002	Low risk	Unclear	Unclear	Unclear	Low risk	Low risk	Low risk

### Study outcomes

#### Depression

**Figure 6:**
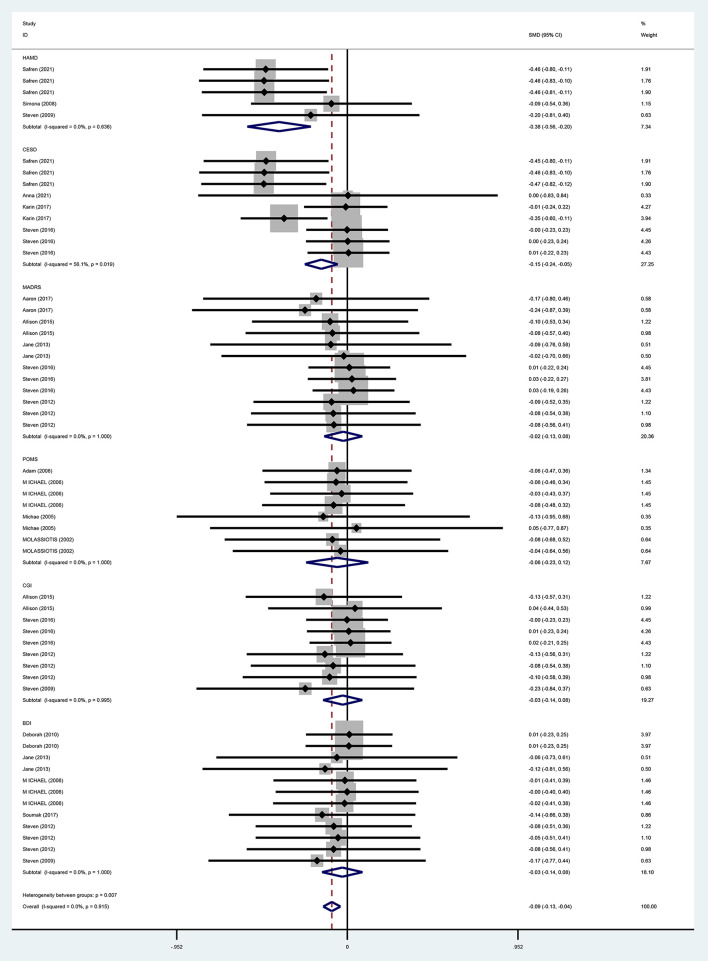
Forest diagram of depressive symptoms scale (SMD).

**Figure 7:**
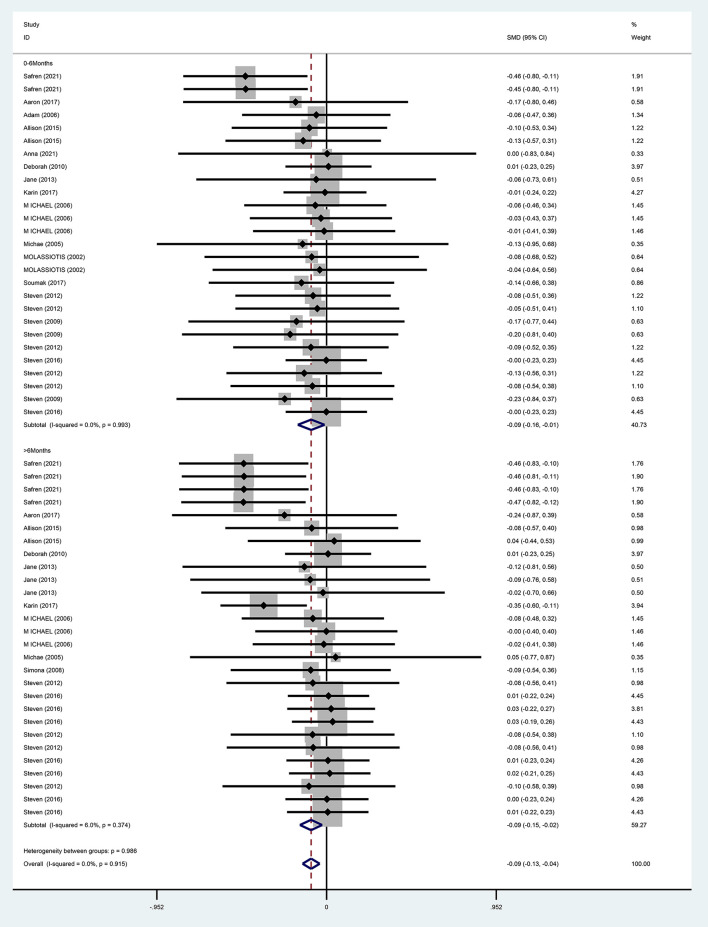
Forest diagram of depressive symptoms period.

For the overall effect on depression, 15 studies (13, 28–37, 39–42) evaluated the effect of CBT on depression in the short term (0–6 months) using BDI, CES-D, HAMD, POMS, CGI, and MADRS, and 11 (13, 28, 30, 32–36, 38, 40, 41) evaluated the effect of CBT on depression in the long term (>6 months, the same below) using BDI, HAMD, POMS, MADRS, CGI, and CES-D, respectively. And the above studies were analyzed in subgroups according to the duration of the intervention effect. Since different scales were adopted for evaluation in each study, the standardized mean difference was used as the effect analysis statistic. Meta-analysis results using the random-effects model showed that the overall difference in depression improvement between the CBT group and control group was statistically significant (SMD = −0.09, 95% CI [−0.13 to −0.04], *P* < 0.001) (Figure 6). Further subgroup analysis indicated that the CBT group improved better than the control group in depression in both follow-up periods, the difference was statistically significant in the short term (0–6 months) (SMD = −0.09, 95% CI [−0.16 to 0.01], *P* = 0.025); in the long term (<6 months), the difference in depression improvement between the two groups was statistically significant (SMD = −0.09, 95% CI [−0.15 to −0.01], *P* = 0.006) (Figure 7).

**Figure 8:**
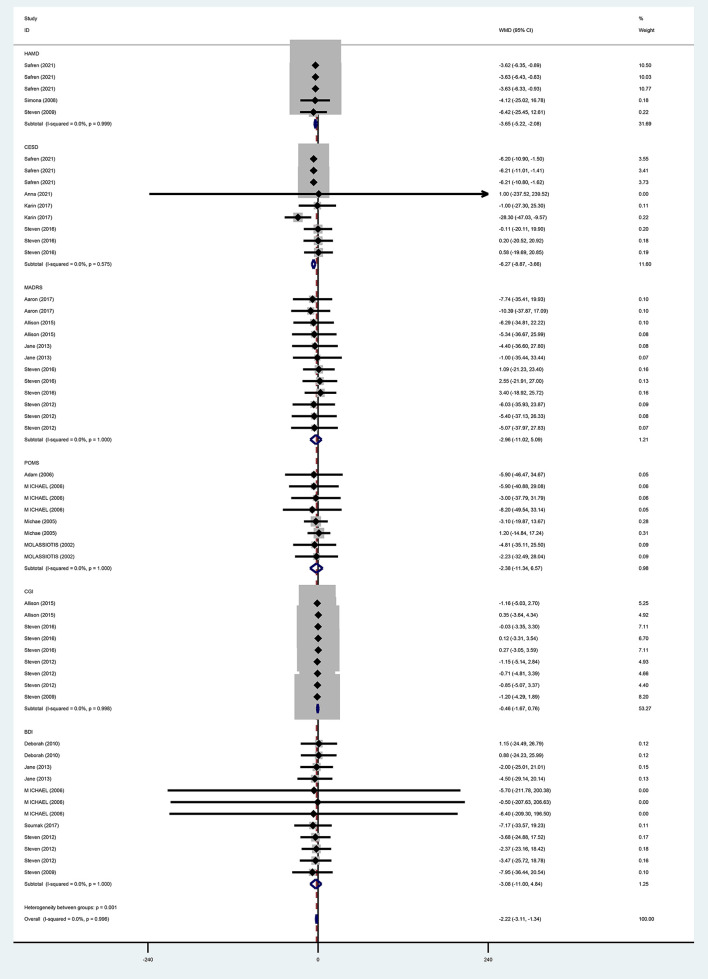
Forest diagram of depressive symptoms scale (WMD).

For the effects of CBT on depression measured by different scales, six studies (13, 31, 35–37, 40) adopted BDI, and meta-analysis results using a fixed-effects model showed that the improvement of depression in the CBT group was not better than that in the control group and that the difference was not statistically significant (WMD = −3.08, 95% CI [−11.00 to 4.84], *P* = 0.446). With three studies (28, 37, 38) using HAMD, meta-analysis results by a fixed-effects model demonstrated that the improvement in depression was better in the CBT group than in the control group and the difference was statistically significant (WMD = −3.65, 95% CI [−5.22 to 2.08], *P* = 0.000). MADRS was used in five studies (28–32), and the meta-analysis using a fixed-effects model showed that CBT improved depression better than the control group, while the difference was not statistically significant (WMD = −2.96, 95% CI [−11.02 to −5.09], *P* = 0.471). POMS was adopted in four (39–42) studies, and the meta-analysis using the fixed-effects model indicated that improvement in depression was better in the CBT group than in the control group, but the difference was not statistically significant (WMD = −2.38, 95% CI [−11.34 to 6.57], *P* = 0.602). With four studies (13, 33, 34, 37) using CGI, meta-analysis results by a fixed-effects model showed that the CBT group improved depression better than the control group, but the difference was not statistically significant (WMD = −0.46, 95% CI [−1.67 to 0.76], *P* = 0.459). Four studies (28–30, 33) adopted CESD, and the meta-analysis using a fixed-effects model showed that the improvement in depression was better in the CBT group than in the control group with a statistically significant difference (WM = −6.27, 95% CI [−8.87 to −3.66], *P* = 0.000) (Figure 8).

#### Adherence

**Figure 9:**
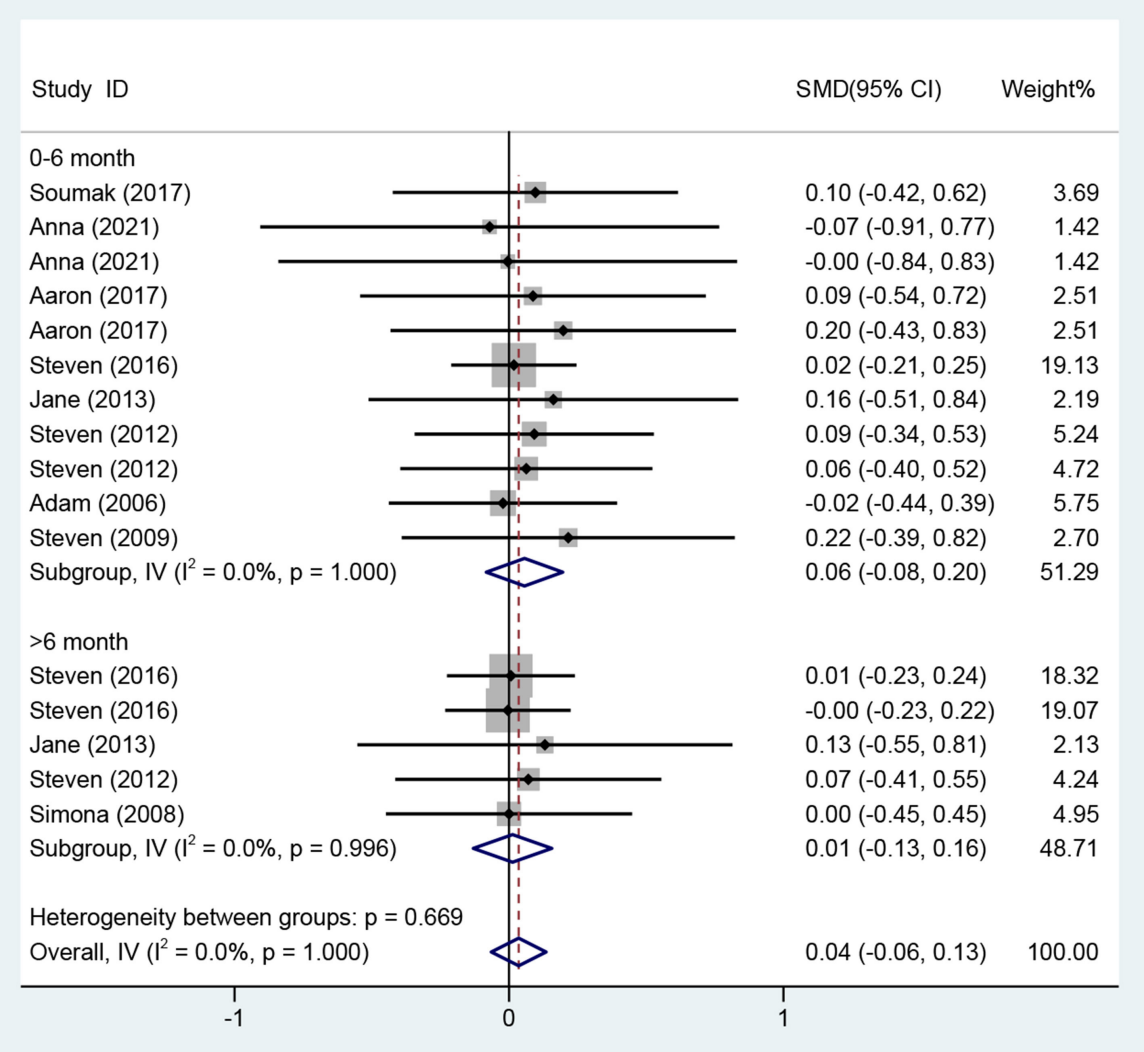
Forest diagram of adherence.

As for the overall effect on ART medication adherence, the meta-analysis results using the fixed-effects model showed that there was no statistically significant difference in the improvement of ART medication adherence in the CBT group compared with the control group (SMD = 0.04, 95% CI [−0.06 to 0.13], *P* = 0.490) (Figure 9).

Regarding the effects on ART medication adherence from 0 to 6 months, eight studies (13, 29–31, 33, 35, 37, 39) evaluated the effects of CBT on ART medication adherence over the period of 0–6 months using different adherence instruments for evaluation, so the standardized mean difference was used as the effect analysis statistic. Meta-analysis of the fixed-effects model revealed no statistically significant difference in the improvement of ART medication adherence in the CBT group compared with the control group (SMD = 0.06, 95% CI [−0.08 to 0.20], *P* = 0.428) (Figure 9).

Four studies (13, 33, 35, 38) on the effects on ART medication adherence over a period of >6 month adopted different evaluation methods, and the standardized mean difference was used as the effect analysis statistic. The meta-analysis results of the fixed-effects model showed no statistically significant difference in the improvement of adherence in the CBT group compared with the control group (SMD = 0.01, 95% CI [−0.13 to 0.16], *P* = 0.860) (Figure 9).

## Discussion

A total of 16 studies with 1,998 patients were included in this systematic evaluation. Two researchers evaluated the risk of bias of included RCTs using the Cochrane Handbook for Systematic Reviewers (version 5.1.0), RCT risk of bias assessment tool. Evaluation indicators include: (1) sequence generation (selection bias); (2) allocation concealment (selection bias); (3) blinding of patients and personnel (performance bias); (4) blinding of outcome assessors (detection bias); (5) incomplete outcome data (attrition bias); (6) elective reporting (reporting bias); (7) other bias. Each indicator contains three levels: low risk, unclear and high risk. Disagreement during the evaluation process may be referred to a third party for determination. The overall quality of the included studies was good, suggesting the reliability of the combined data in this meta-analysis (Table 2).

Meta-analysis results suggested that CBT could improve depressive symptoms in PLWH. Meta-analysis results from this systematic evaluation of studies indicated that CBT improved depression in PLWH in general, with more significant long-term (>6 months) effects than short-term (0–6 months). This result is consistent with a study by Tobin et al. (30), which administered 3-month CBT to 165 PLWH (6 ≤ CES-D ≤ 40) in a randomized controlled trial with nine group sessions and one individual session. A comparison of baseline, 6 and 12 months CES-D scores revealed that CES-D depression scores were lower than baseline levels in both groups during follow-up, and the CES-D score of 17.1 at the 12-month follow-up was less than the 19.2 at 6 months, indicating a good long-term benefit of CBT. There is evidence (43) that 6 months is a critical period for achieving significant efficacy of antiviral therapy in PLWH. Among the disease factors, CD4 (Cluster of Differentiation 4) + T lymphocytes <200/μl and disease stage of AIDS are risk factors for depression in PLWH, suggesting an association between disease severity and depression. StuWMDdies have found that the decrease of CD4 + T lymphocytes will aggravate the depressive symptoms of PLWH (44), while PLWH in a depressed state have a significantly greater decline in CD4 + T lymphocytes than those without depression (45), and the two are correlated and interact with each other. PLWH phase are at increased risk of depression with the onset of various somatic symptoms and opportunistic infections (46, 47). Hence, the more pronounced improvement in depression over 6 months in this systematic analysis may also be related to the fact that PLWH have undergone 6 months of antiretroviral therapy.

When studies were included in the subgroup analysis by different measurement scales, CBT improved depression in PLWH when depression was evaluated by HAMD, MADRS, CES-D, POMS, and CGI, while CBT did not show an effect of improving depression in PLWH when depression was evaluated by BDI. The slight differences in the above results may be related to some errors in the evaluation of depression by different scales, or the adoption of a group-based treatment model in the CBT group in some of the studies. A meta-analysis showed that CBT-based group psychological interventions had a small effect on measures of depression in PLWH, lasting until the end of the 15-month follow-up; the Beck Depression Inventory (BDI) was used in most previous studies, with a maximum score of 63, and at the end of the intervention, the mean score for the treatment group was only 1.4 points lower than that of the control group (48). This may be related to the fact that group therapy cannot take into account the cultural background of every participant and the particularity of the disease makes PLWH less active in participating. For individual treatment, the intervenor can choose a more targeted treatment plan according to the different cultural and personality characteristics, interests and hobbies of each patient, so as to make the treatment effect more significant.

CBT aims to help clients better cope with their emotional distress by changing their irrational thinking and behavior patterns (49). When conducting CBT, the medical staff and clients work jointly to identify the problem to be solved, develop solutions for different situations, make decisions on what to adopt, and formulate implementation plans (13). Thus, CBT helps clients to better manage the problems they face and thereby improve depression. The long-term (>6 months) effects of CBT on PLWH depression are more significant than short-term (0–6 months), indicating that it takes time to maintain the effects of CBT interventions.

The results of this systematic evaluation suggest that the effects of CBT on improving antiretroviral therapy (ART) medication adherence in PLWH are unclear. This may be due to the fact that the medication taking behavior of PLWH is influenced by various factors, such as the patient's personal factors, disease factors, psychosocial factors, and the doctor-patient relationship, all of which can affect the patient's adherence (50). Furthermore, as no uniform conclusion has been reached on the factors influencing ART adherence among PLWH due to differences in social environment, adherence assessment indicators, study population, and study methods (51, 52), the improvement in depressive symptoms may have had only a partial effect on ART medication adherence, thereby resulting in a non-significant overall effect. Meta-analysis results based on eight short-term effect RCTs and four long-term effect RCTs showed that the long-term (>6 months) effect of CBT on improving antiretroviral therapy (ART) medication adherence in PLWH was better than the short-term (0–6 months) effect. This is consistent with the findings of a meta-analysis in China suggesting that CBT has a long-term favorable effect on improving medication adherence and PLWH (53).

The meta-analysis by Dimatteo et al. showed that depressed clients were three times more likely to not comply with medical treatment recommendations compared with non-depressed patients (54). Therefore, depression is one of the important risk factors for treatment non-adherence in PLWH. The above results may be attributed to the fact that CBT, as a psychological intervention mode, requires a longer period of intervention to prevent PLWH from gradually stopping changing, so as to improve clients self-management ability and thus ART medication adherence (55). The maximum follow-up for ART medication adherence included in this systematic evaluation was 12 months, and longer interventions may still be needed.

## Limitations

This systematic review of included studies may have some heterogeneity in sample and methodology such as sample size, article quality, and outcome measures; the content, starting time, frequency and duration of intervention programs varied greatly; there were also great differences in the evaluation methods and scales of outcome indicators. Meanwhile, there may be publication bias due to incomplete inclusion of literature as only published literature was searched.

## Conclusions

CBT is an effective treatment for depression, and in this systematic review, we have found that CBT has a potential improvement effect on PLWH combined with depression, but the improvement of ART medication adherence needs further study. Therefore, policymakers should provide policies and funding to support CBT interventions for PLWH depression and ART medication adherence, and health care providers should consider CBT in PLWH to improve depression in PLWH. The short-term effect of CBT on improving ART medication adherence in PLWH remains unclear, and thus it is recommended that policymakers and healthcare providers invest in multi-center RCTs with large sample sizes to confirm the effect of CBT on improving medication antiretroviral therapy (ART) medication adherence in PLWH.

## Data availability statement

The original contributions presented in the study are included in the article/Supplementary material, further inquiries can be directed to the corresponding author.

## Author contributions

KQ and JZ wrote the main manuscript, fully participated in all analyses, and contributed to the study concept and design. LL and YC participated in literature search, data extraction, and quality assessment. All authors read and approved the final manuscript.

## Funding

This work was supported by the scientific research start-up funds (Grant No. DC2200002888).

## Conflict of interest

The authors declare that the research was conducted in the absence of any commercial or financial relationships that could be construed as a potential conflict of interest.

## Publisher's note

All claims expressed in this article are solely those of the authors and do not necessarily represent those of their affiliated organizations, or those of the publisher, the editors and the reviewers. Any product that may be evaluated in this article, or claim that may be made by its manufacturer, is not guaranteed or endorsed by the publisher.
