# Supplementary material for: Effects of cognitive behavioral therapy on improving depressive symptoms and increasing adherence to antiretroviral medication in people with HIV
Source: Front Psychiatry. 2022 Nov 9;13:990994. doi: 10.3389/fpsyt.2022.990994 (PMC9682157; doi:10.3389/fpsyt.2022.990994)
Supplement: Supplementary file 1 [file Table_1.DOCX]

**Appendix** S1: Search strategy

**PubMed**

18 ((("Cognitive Behavioral Therapy"[Mesh]) OR ((((((((((((((((Cognitive Behavioral Therapy[Title/Abstract]) OR (Behavioral Therap*, Cognitive[Title/Abstract])) OR (Behaviour Therap*, Cognitive[Title/Abstract])) OR (Behavior Therapy, Cognitive[Title/Abstract])) OR (Cognitive Behavioral Therapies[Title/Abstract])) OR (Cognitive Therap*[Title/Abstract])) OR (Cognitive Behaviour Therap*[Title/Abstract])) OR (Cognitive Psychotherap*[Title/Abstract])) OR (Cognition Therap*[Title/Abstract])) OR (Cognitive Behavior Therap*[Title/Abstract])) OR (Therap*, Cognitive Behavioral[Title/Abstract])) OR (Therap*, Cognitive[Title/Abstract])) OR (Therap*, Cognitive Behaviour[Title/Abstract])) OR (Therap*, Cognition[Title/Abstract])) OR (Therap*, Cognitive Behavior[Title/Abstract])) OR (Psychotherap*, Cognitive[Title/Abstract]))) AND ((("HIV"[Mesh]) OR (((((((((((((((HIV[Title/Abstract]) OR (Human Immunodeficiency Virus*[Title/Abstract])) OR (Human T Cell Lymphotropic Virus Type III[Title/Abstract])) OR (Human T Cell Leukemia Virus Type III[Title/Abstract])) OR (Human T Lymphotropic Virus Type III[Title/Abstract])) OR (Immunodeficiency Virus*, Human[Title/Abstract])) OR (Virus*, Human Immunodeficiency[Title/Abstract])) OR (LAV-HTLV-III[Title/Abstract])) OR (Lymphadenopathy-Associated Virus*[Title/Abstract])) OR (Virus*, Lymphadenopathy-Associated[Title/Abstract])) OR (AIDS Virus*[Title/Abstract])) OR (Virus*, AIDS[Title/Abstract])) OR (Acquired Immune Deficiency Syndrome Virus[Title/Abstract])) OR (Acquired Immunodeficiency Syndrome Virus[Title/Abstract])) OR (HTLV-III[Title/Abstract]))) OR (("Acquired Immunodeficiency Syndrome"[Mesh]) OR ((((((((((Acquired Immunodeficiency Syndrome[Title/Abstract]) OR (Acquired Immune Deficiency Syndrome*[Title/Abstract])) OR (Acquired Immuno Deficiency Syndrome*[Title/Abstract])) OR (Acquired Immunodeficiency Syndromes[Title/Abstract])) OR (AIDS[Title/Abstract])) OR (Immunologic Deficiency Syndrome, Acquired[Title/Abstract])) OR (Immuno-Deficiency Syndrome*, Acquired[Title/Abstract])) OR (Immunodeficiency Syndrome*, Acquired[Title/Abstract])) OR (Syndrome*, Acquired Immuno-Deficiency[Title/Abstract])) OR (Syndrome*, Acquired Immunodeficiency[Title/Abstract]))))) AND ((("Depression"[Mesh]) OR (((((Depression[Title/Abstract]) OR (Depressive Symptom*[Title/Abstract])) OR (Symptom*, Depressive[Title/Abstract])) OR (Emotional Depression[Title/Abstract])) OR (Depression, Emotiona[Title/Abstract]))) OR (("Depressive Disorder"[Mesh]) OR ((((((((((((((Depressive Disorder[Title/Abstract]) OR (Depressive Disorders[Title/Abstract])) OR (Disorde*, Depressive[Title/Abstract])) OR (Neuros*, Depressive[Title/Abstract])) OR (Depressive Neuros*[Title/Abstract])) OR (Depression*, Endogenous[Title/Abstract])) OR (Endogenous Depression*[Title/Abstract])) OR (Depressive Syndrome*[Title/Abstract])) OR (Syndrome*, Depressive[Title/Abstract])) OR (Depression*, Neurotic[Title/Abstract])) OR (Neurotic Depression*[Title/Abstract])) OR (Melancholia*[Title/Abstract])) OR (Unipolar Depression*[Title/Abstract])) OR (Depression*, Unipolar[Title/Abstract]))))

17 (("Depression"[Mesh]) OR (((((Depression[Title/Abstract]) OR (Depressive Symptom*[Title/Abstract])) OR (Symptom*, Depressive[Title/Abstract])) OR (Emotional Depression[Title/Abstract])) OR (Depression, Emotiona[Title/Abstract]))) OR (("Depressive Disorder"[Mesh]) OR ((((((((((((((Depressive Disorder[Title/Abstract]) OR (Depressive Disorders[Title/Abstract])) OR (Disorde*, Depressive[Title/Abstract])) OR (Neuros*, Depressive[Title/Abstract])) OR (Depressive Neuros*[Title/Abstract])) OR (Depression*, Endogenous[Title/Abstract])) OR (Endogenous Depression*[Title/Abstract])) OR (Depressive Syndrome*[Title/Abstract])) OR (Syndrome*, Depressive[Title/Abstract])) OR (Depression*, Neurotic[Title/Abstract])) OR (Neurotic Depression*[Title/Abstract])) OR (Melancholia*[Title/Abstract])) OR (Unipolar Depression*[Title/Abstract])) OR (Depression*, Unipolar[Title/Abstract])))

16 ("Depressive Disorder"[Mesh]) OR ((((((((((((((Depressive Disorder[Title/Abstract]) OR (Depressive Disorders[Title/Abstract])) OR (Disorde*, Depressive[Title/Abstract])) OR (Neuros*, Depressive[Title/Abstract])) OR (Depressive Neuros*[Title/Abstract])) OR (Depression*, Endogenous[Title/Abstract])) OR (Endogenous Depression*[Title/Abstract])) OR (Depressive Syndrome*[Title/Abstract])) OR (Syndrome*, Depressive[Title/Abstract])) OR (Depression*, Neurotic[Title/Abstract])) OR (Neurotic Depression*[Title/Abstract])) OR (Melancholia*[Title/Abstract])) OR (Unipolar Depression*[Title/Abstract])) OR (Depression*, Unipolar[Title/Abstract]))

15 (((((((((((((Depressive Disorder[Title/Abstract]) OR (Depressive Disorders[Title/Abstract])) OR (Disorde*, Depressive[Title/Abstract])) OR (Neuros*, Depressive[Title/Abstract])) OR (Depressive Neuros*[Title/Abstract])) OR (Depression*, Endogenous[Title/Abstract])) OR (Endogenous Depression*[Title/Abstract])) OR (Depressive Syndrome*[Title/Abstract])) OR (Syndrome*, Depressive[Title/Abstract])) OR (Depression*, Neurotic[Title/Abstract])) OR (Neurotic Depression*[Title/Abstract])) OR (Melancholia*[Title/Abstract])) OR (Unipolar Depression*[Title/Abstract])) OR (Depression*, Unipolar[Title/Abstract])

14 "Depressive Disorder"[Mesh]

13 ("Depression"[Mesh]) OR (((((Depression[Title/Abstract]) OR (Depressive Symptom*[Title/Abstract])) OR (Symptom*, Depressive[Title/Abstract])) OR (Emotional Depression[Title/Abstract])) OR (Depression, Emotiona[Title/Abstract]))

12 ((((Depression[Title/Abstract]) OR (Depressive Symptom*[Title/Abstract])) OR (Symptom*, Depressive[Title/Abstract])) OR (Emotional Depression[Title/Abstract])) OR (Depression, Emotiona[Title/Abstract])

11 "Depression"[Mesh]

10 (("HIV"[Mesh]) OR (((((((((((((((HIV[Title/Abstract]) OR (Human Immunodeficiency Virus*[Title/Abstract])) OR (Human T Cell Lymphotropic Virus Type III[Title/Abstract])) OR (Human T Cell Leukemia Virus Type III[Title/Abstract])) OR (Human T Lymphotropic Virus Type III[Title/Abstract])) OR (Immunodeficiency Virus*, Human[Title/Abstract])) OR (Virus*, Human Immunodeficiency[Title/Abstract])) OR (LAV-HTLV-III[Title/Abstract])) OR (Lymphadenopathy-Associated Virus*[Title/Abstract])) OR (Virus*, Lymphadenopathy-Associated[Title/Abstract])) OR (AIDS Virus*[Title/Abstract])) OR (Virus*, AIDS[Title/Abstract])) OR (Acquired Immune Deficiency Syndrome Virus[Title/Abstract])) OR (Acquired Immunodeficiency Syndrome Virus[Title/Abstract])) OR (HTLV-III[Title/Abstract]))) OR (("Acquired Immunodeficiency Syndrome"[Mesh]) OR ((((((((((Acquired Immunodeficiency Syndrome[Title/Abstract]) OR (Acquired Immune Deficiency Syndrome*[Title/Abstract])) OR (Acquired Immuno Deficiency Syndrome*[Title/Abstract])) OR (Acquired Immunodeficiency Syndromes[Title/Abstract])) OR (AIDS[Title/Abstract])) OR (Immunologic Deficiency Syndrome, Acquired[Title/Abstract])) OR (Immuno-Deficiency Syndrome*, Acquired[Title/Abstract])) OR (Immunodeficiency Syndrome*, Acquired[Title/Abstract])) OR (Syndrome*, Acquired Immuno-Deficiency[Title/Abstract])) OR (Syndrome*, Acquired Immunodeficiency[Title/Abstract])))

9 ("Acquired Immunodeficiency Syndrome"[Mesh]) OR ((((((((((Acquired Immunodeficiency Syndrome[Title/Abstract]) OR (Acquired Immune Deficiency Syndrome*[Title/Abstract])) OR (Acquired Immuno Deficiency Syndrome*[Title/Abstract])) OR (Acquired Immunodeficiency Syndromes[Title/Abstract])) OR (AIDS[Title/Abstract])) OR (Immunologic Deficiency Syndrome, Acquired[Title/Abstract])) OR (Immuno-Deficiency Syndrome*, Acquired[Title/Abstract])) OR (Immunodeficiency Syndrome*, Acquired[Title/Abstract])) OR (Syndrome*, Acquired Immuno-Deficiency[Title/Abstract])) OR(Syndrome*,Acquired Immunodeficiency[Title/Abstract]))

8 (((((((((Acquired Immunodeficiency Syndrome[Title/Abstract]) OR (Acquired Immune Deficiency Syndrome*[Title/Abstract])) OR (Acquired Immuno Deficiency Syndrome*[Title/Abstract])) OR (Acquired Immunodeficiency Syndromes[Title/Abstract])) OR (AIDS[Title/Abstract])) OR (Immunologic Deficiency Syndrome, Acquired[Title/Abstract])) OR (Immuno-Deficiency Syndrome*, Acquired[Title/Abstract])) OR (Immunodeficiency Syndrome*, Acquired[Title/Abstract])) OR (Syndrome*, Acquired Immuno-Deficiency[Title/Abstract])) OR (Syndrome*, Acquired Immunodeficiency[Title/Abstract])

7 "Acquired Immunodeficiency Syndrome"[Mesh]

6 ("HIV"[Mesh]) OR (((((((((((((((HIV[Title/Abstract]) OR (Human Immunodeficiency Virus*[Title/Abstract])) OR (Human T Cell Lymphotropic Virus Type III[Title/Abstract])) OR (Human T Cell Leukemia Virus Type III[Title/Abstract])) OR (Human T Lymphotropic Virus Type III[Title/Abstract])) OR (Immunodeficiency Virus*, Human[Title/Abstract])) OR (Virus*, Human Immunodeficiency[Title/Abstract])) OR (LAV-HTLV-III[Title/Abstract])) OR (Lymphadenopathy-Associated Virus*[Title/Abstract])) OR (Virus*, Lymphadenopathy-Associated[Title/Abstract])) OR (AIDS Virus*[Title/Abstract])) OR (Virus*, AIDS[Title/Abstract])) OR (Acquired Immune Deficiency Syndrome Virus[Title/Abstract])) OR (Acquired Immunodeficiency Syndrome Virus[Title/Abstract])) OR (HTLV-III[Title/Abstract]))

5 ((((((((((((((HIV[Title/Abstract]) OR (Human Immunodeficiency Virus*[Title/Abstract])) OR (Human T Cell Lymphotropic Virus Type III[Title/Abstract])) OR (Human T Cell Leukemia Virus Type III[Title/Abstract])) OR (Human T Lymphotropic Virus Type III[Title/Abstract])) OR (Immunodeficiency Virus*, Human[Title/Abstract])) OR (Virus*, Human Immunodeficiency[Title/Abstract])) OR (LAV-HTLV-III[Title/Abstract])) OR (Lymphadenopathy-Associated Virus*[Title/Abstract])) OR (Virus*, Lymphadenopathy-Associated[Title/Abstract])) OR (AIDS Virus*[Title/Abstract])) OR (Virus*, AIDS[Title/Abstract])) OR (Acquired Immune Deficiency Syndrome Virus[Title/Abstract])) OR (Acquired Immunodeficiency Syndrome Virus[Title/Abstract])) OR (HTLV-III[Title/Abstract])

4 "HIV"[Mesh]

3 ("Cognitive Behavioral Therapy"[Mesh]) OR ((((((((((((((((Cognitive Behavioral Therapy[Title/Abstract]) OR (Behavioral Therap*, Cognitive[Title/Abstract])) OR (Behaviour Therap*, Cognitive[Title/Abstract])) OR (Behavior Therapy, Cognitive[Title/Abstract])) OR (Cognitive Behavioral Therapies[Title/Abstract])) OR (Cognitive Therap*[Title/Abstract])) OR (Cognitive Behaviour Therap*[Title/Abstract])) OR (Cognitive Psychotherap*[Title/Abstract])) OR (Cognition Therap*[Title/Abstract])) OR (Cognitive Behavior Therap*[Title/Abstract])) OR (Therap*, Cognitive Behavioral[Title/Abstract])) OR (Therap*, Cognitive[Title/Abstract])) OR (Therap*, Cognitive Behaviour[Title/Abstract])) OR (Therap*, Cognition[Title/Abstract])) OR (Therap*, Cognitive Behavior[Title/Abstract])) OR (Psychotherap*, Cognitive[Title/Abstract]))

2 (((((((((((((((Cognitive Behavioral Therapy[Title/Abstract]) OR (Behavioral Therap*, Cognitive[Title/Abstract])) OR (Behaviour Therap*, Cognitive[Title/Abstract])) OR (Behavior Therapy, Cognitive[Title/Abstract])) OR (Cognitive Behavioral Therapies[Title/Abstract])) OR (Cognitive Therap*[Title/Abstract])) OR (Cognitive Behaviour Therap*[Title/Abstract])) OR (Cognitive Psychotherap*[Title/Abstract])) OR (Cognition Therap*[Title/Abstract])) OR (Cognitive Behavior Therap*[Title/Abstract])) OR (Therap*, Cognitive Behavioral[Title/Abstract])) OR (Therap*, Cognitive[Title/Abstract])) OR (Therap*, Cognitive Behaviour[Title/Abstract])) OR (Therap*, Cognition[Title/Abstract])) OR (Therap*, Cognitive Behavior[Title/Abstract])) OR (Psychotherap*, Cognitive[Title/Abstract])

1 "Cognitive Behavioral Therapy"[Mesh]
